# Potential feasibility of a novel over‐the‐wire microelectrode catheter for recording coronary sinus electrograms in patients with cardiac resynchronization therapy devices

**DOI:** 10.1002/joa3.12876

**Published:** 2023-05-28

**Authors:** Masato Okada, Naoko Miyazaki, Koji Tanaka, Yusuke Ikada, Nobuaki Tanaka

**Affiliations:** ^1^ Cardiovascular Center Sakurabashi Watanabe Hospital Osaka Japan

**Keywords:** atrial fibrillation, cardiac resynchronization therapy, catheter ablation, microelectrode, tachycardias

## Abstract

The placement of an electrode catheter into the coronary sinus (CS) is important for differentiating multiple atrial tachycardias (ATs). Based on its high selective performance in placement into small veins, inserting a novel over‐the‐wire 2.7Fr microcatheter (EPstar Fix AIV; Japan Lifeline) into the CS may be feasible even in patients implanted with a cardiac resynchronization therapy device.

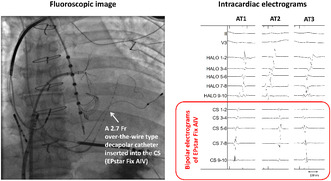

## 1 DESCRIPTIONS

The placement of an electrode catheter into the coronary sinus (CS) is important for electrophysiological studies. The patterns and timing of the CS activation provide a rapid stratification of the most likely macroreentrant atrial tachycardias (ATs).^1^ Overdrive pacing from the CS catheter can help differentiate whether the AT is of left or right atrial origin.^2^ Local electrograms recorded from the CS are often used as a reference in three‐dimensional mapping systems.^3^ Despite the above benefits, the insertion of CS catheters is discouraged in patients implanted with cardiac resynchronization therapy (CRT) devices due to the risk of impairment of the left ventricular (LV) lead.

**FIGURE 1:**
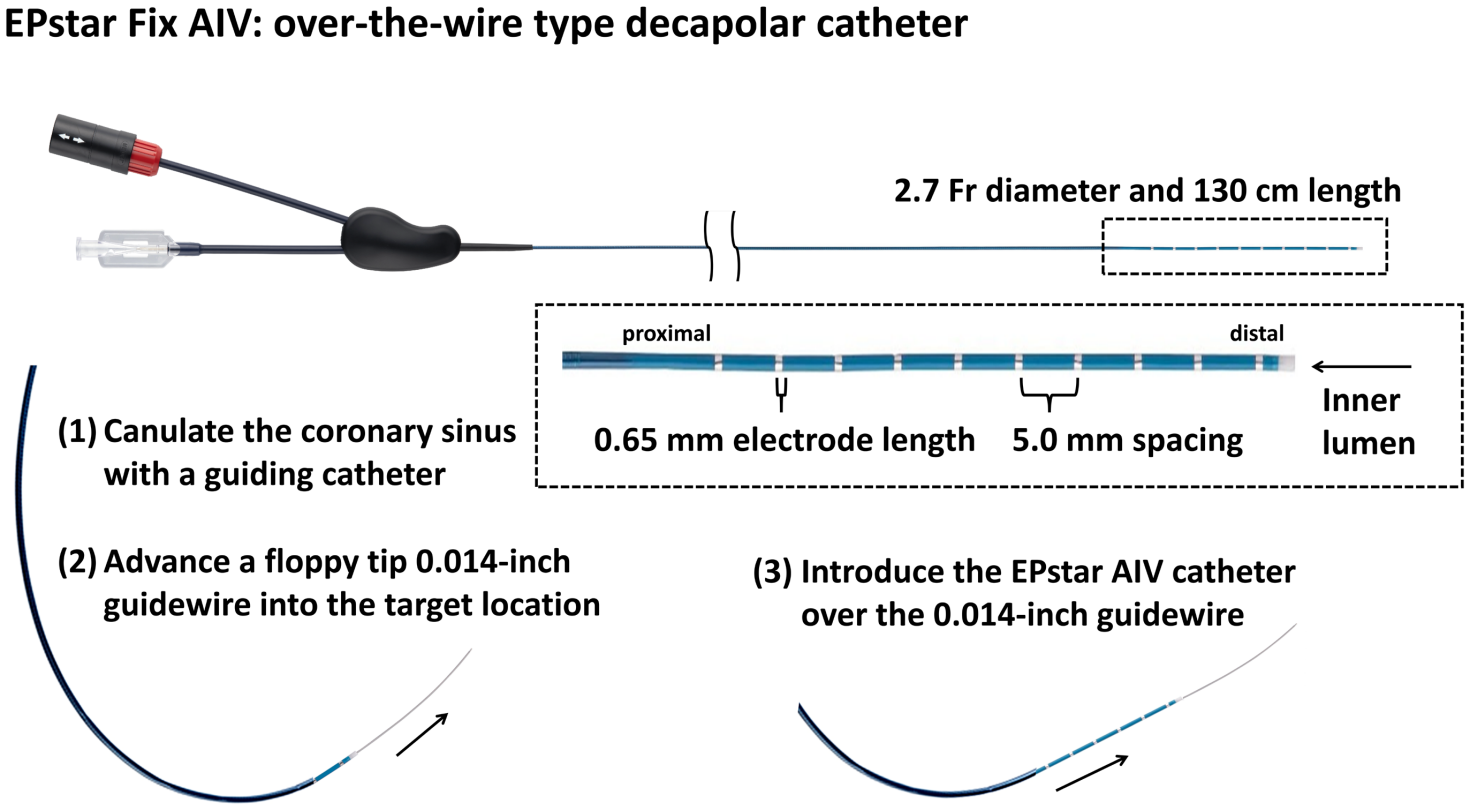
The EPstar Fix AIV (Japan Lifeline) is a 2.7‐Fr over‐the‐wire type microcatheter with 10 electrodes, each measuring 0.65 mm in length and arranged at 5‐mm intervals. This device features an inner lumen that allows the insertion of a 0.014‐inch guidewire. The basic steps for using this microcatheter are as follows: (1) canulate the coronary sinus with a guiding catheter, (2) advance a 0.014‐inch guidewire to the intended target location, and (3) introduce the 2.7‐Fr microcatheter over the guidewire. The microcatheter can record the local electrograms without being affected by far‐field activation in bipolar mapping. Because it is possible to evaluate multiple consecutive bipolar electrograms simultaneously, this microcatheter can help us diagnose atrial tachycardias.

Recently, a novel over‐the‐wire 2.7‐Fr microcatheter (EPstar Fix AIV; Japan Lifeline) has become available (Figure 1). Because of its small diameter and over‐the‐wire type structure, inserting it into the CS distal veins (i.e., anterior interventricular vein or communicating vein) is feasible and can be performed safely, particularly when treating outflow tract ventricular arrhythmias.^4^ Based on its high selective performance in placement into small veins, we hypothesized that inserting the microcatheter into the CS would be feasible even in patients equipped with CRT devices.

**FIGURE 2:**
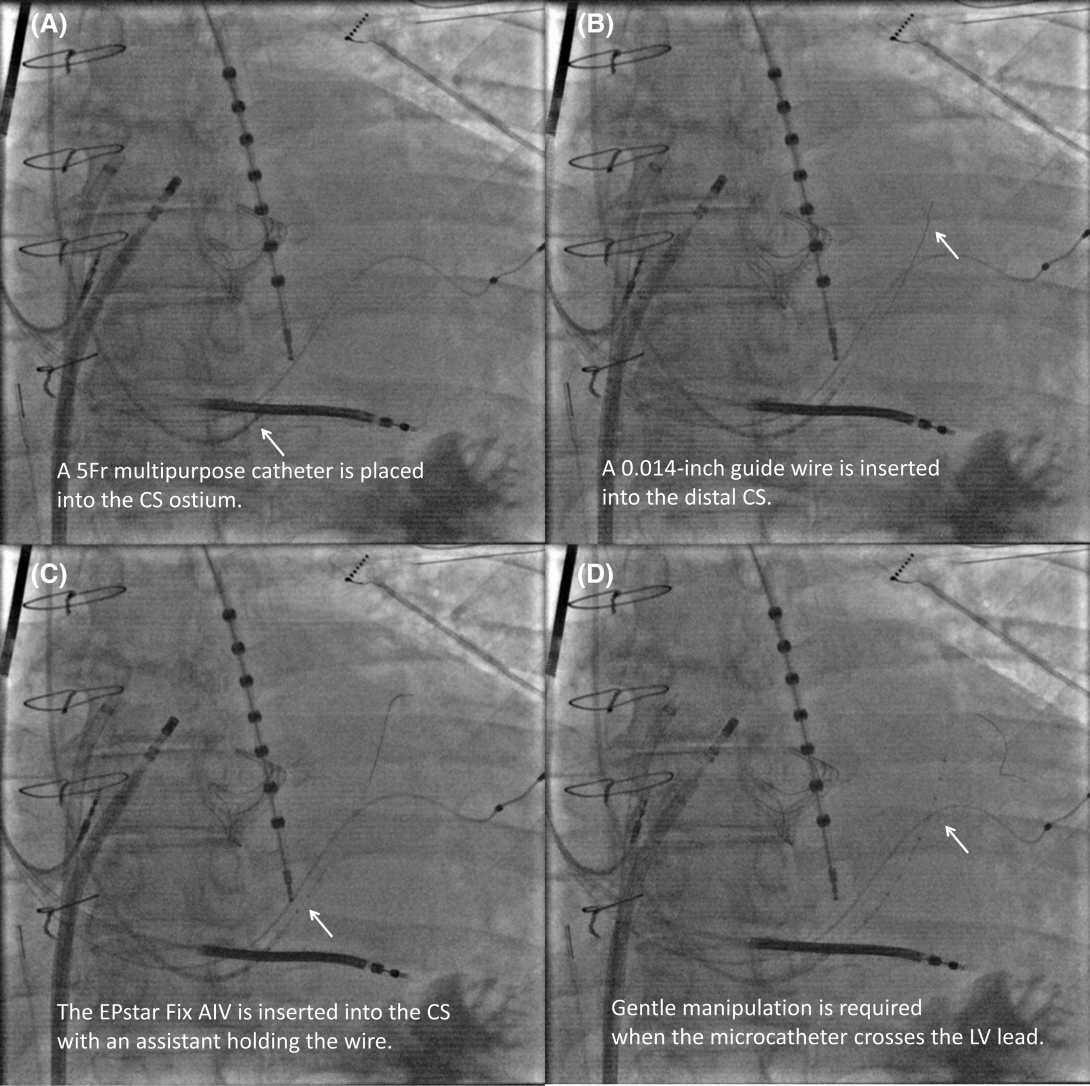
Fluoroscopic images obtained during the catheter insertion procedure. Instead of a 6‐Fr decapolare catheter with an inner lumen (EPstar Fixed CS; Japan Lifeline), a 5‐Fr multipurpose catheter is used as a guide for inserting the microcatheter to prevent any unexpected injury to the left ventricular lead. First, a 0.014‐inch guidewire and the EPstar Fix AIV are introduced into the 5‐Fr multipurpose catheter outside the body. The 5‐Fr multipurpose catheter is then advanced into the right atrium with the guidewire ahead of the device. Subsequently, the guidewire and EPstar Fix AIV are stored within the 5‐Fr multipurpose catheter, which is then navigated into the CS ostium using its curvature (A). A wire‐guided cannulation is an option in difficult cases. Next, the 0.014‐inch guidewire is advanced into the distal CS while ensuring that the tip of the guidewire is not repelled by venous stenosis or occlusion (B). Finally, the EPstar Fix AIV is advanced along the wire while an assistant holds it (C). When the microcatheter crosses the left ventricular lead, care should be taken not to dislodge the multipurpose catheter by counterforce and ensure that there is no resistance (D).

To date, we have used the microcatheter in four patients with CRT devices in the setting of atrial fibrillation and AT ablation. Using a 5‐Fr multipurpose catheter as a guide to the CS ostium, we inserted the over‐the‐wire microcatheter into the CS in all four patients (Figure 2; Video S1). The quality of the local electrograms and pacing threshold were similar to those of the conventional multielectrode catheter. Fortunately, no complications have occurred yet (e.g., lead dislodgement, increased pacing threshold, or decreased local electrograms) (Table S1), which might be due to the procedure being performed in cases where the LV lead had been implanted for more than 6 months. Considering the individual CS anatomies^5^ and various LV lead placement sites, further validation and gentle manipulation are necessary. Nevertheless, inserting this novel microelectrode catheter into the CS may be feasible to help diagnose complex ATs in patients with CRT devices.

## FUNDING INFORMATION

This research did not receive any specific grant from funding agencies in the public, commercial, or not‐for‐profit sectors.

## CONFLICT OF INTEREST STATEMENT

The authors have nothing to disclose.

## APPROVAL OF THE RESEARCH PROTOCOL

Approval was obtained from the local ethics committee (IRB #23–24).

## INFORMED CONSENT

Patient consent for publication was obtained.

## REGISTRY AND REGISTRATION NO. OF THE STUDY/TRIAL

Not applicable.

## ANIMAL STUDIES

Not applicable.

## Supporting information


Data S1.
