# Supplementary material for: Potential feasibility of a novel over‐the‐wire microelectrode catheter for recording coronary sinus electrograms in patients with cardiac resynchronization therapy devices
Source: J Arrhythm. 2023 May 28;39(4):645–7. doi: 10.1002/joa3.12876 (PMC10407166; doi:10.1002/joa3.12876)
Supplement: Supplementary file 1 — Data S1. [file JOA3-39-645-s001.zip › JOA3_12876-sup-0001-TableS1.docx]

**Supplementary Table 1.**

| **Case** | **Age, years** | **Sex** | **Target arrhythmia** | **Etiology** | **Device** | **LVEF (%)** | **Lead age* (months)** | **Lead impedance (Ω)** | | **Pacing threshold (mV/0.40 ms)** | | **Bipolar voltage (mV)** | |
| --- | --- | --- | --- | --- | --- | --- | --- | --- | --- | --- | --- | --- | --- |
|  |  |  |  |  |  |  |  | **Pre-** | **Post** | **Pre-** | **Post-** | **Pre-** | **Post** |
| # 1 | 57 | Male | AT post-AF ablation | DCM | CRT-D | 23 | 42 | 418 | 399 | 0.375 | 0.375 | 6.0 | 6.9 |
| # 2 | 75 | Male | AT post-AF ablation | CP | CRT-P | 33 | 6 | 507 | 520 | 1.90 | 2.20 | 8.5 | 8.5 |
| # 3 | 72 | Male | Persistent AF | ICM | CRT-D | 18 | 35 | 521 | 442 | 1.60 | 1.60 | 21.1 | 19.0 |
| # 4 | 69 | Male | Persistent AF | DCM | CRT-D | 41 | 129 | 437 | 418 | 2.00 | 2.00 | NA† | NA† |

*Lead age describes the length of time since the LV lead placement.

†Due to complete atrioventricular block, the bipolar voltage was not available in this case.

AF, atrial fibrillation; AT, atrial tachycardia; CP, constructive pericarditis; CRT-D, cardiac resynchronization therapy defibrillator; CRT-P, cardiac resynchronization therapy pacing; DCM, dilated cardiomyopathy; ICM, ischemic cardiomyopathy; LV, left ventricular; LVEF, left ventricular ejection fraction; NA, not available
